# Severe necrotizing soft tissue infections (SENSEI) study: Protocol for a multi-centered audit

**DOI:** 10.1016/j.jpra.2025.11.016

**Published:** 2025-11-15

**Authors:** R. Yoshimura, T. Hughes, M. Carter, C. Sethu, J.C.R. Wormald

**Affiliations:** a York and Scarborough Teaching Hospitals NHS Foundation Trust, York, UK; b Oxford University Hospitals NHS Foundation Trust, Oxford, UK; c Nuffield Department of Orthopaedics Rheumatology and Musculoskeletal Sciences, University of Oxford, Oxford, UK

**Keywords:** Severe necrotizing soft tissue infections, Sepsis, Necrotizing fasciitis, Fournier’s gangrene

## Abstract

**Background:**

Severe soft tissue infections are surgical emergencies typically accompanied by sepsis. Early recognition and management optimizes patient outcomes. Single-centered studies on these infections are limited due to their rare incidence.

**Materials & methods:**

We propose a protocol for the SENSEI (Severe Necrotizing Soft Tissue Infections) study, a multi-centered audit across the United Kingdom to assess current clinical practice for treating severe soft tissue infections in adult and paediatric populations. This will be compared with standards predetermined by the Surviving Sepsis Campaign and Sepsis 6. This audit will follow a trainee collaborative model, where doctors in surgery and ICU are invited to participate by the Reconstructive Surgery Trials Network and Paediatric Critical Care Society Audit Network. Each site will have a local lead, and at least one collaborator each from surgical and intensive care teams. Data collected will include presentation, investigations, and management of severe soft tissue infections.

**Ethics & dissemination:**

As the SENSEI study is an audit and not research, approval is not required from a NHS research ethics committee. Findings of the study will be reported in scientific meetings and peer-reviewed journals.

## Introduction

Severe necrotizing soft tissue infections (SSTIs) are a group of life-threatening infections, with reported mortality rates as high as 40 %.^1^ There are estimated to be 500 new cases every year with 0.4 to 0.53 cases per 100,000 population.^2^^,^^3^ They are surgical emergencies, characterized by rapidly progressive necrosis of subcutaneous tissue, fascia or muscle, accompanied by sepsis. In addition to mortality, SSTIs can have a long-term psychosocial impact on both patients and their families.^4^ Prompt surgical management and broad-spectrum antibiotics are essential for treating SSTIs. Surgery involves complete debridement of infected and necrotic tissue to limit the spread of the infection.^5^^,^^6^ In extensive upper and lower limb infections, this may involve amputation. Early debridement leads to reduced mortality, fewer number of surgical debridements needed, a lower risk of septic shock and multi-organ failure, and a shorter hospital stay.^7^, ^8^, ^9^, ^10^

While early diagnosis and management can minimize mortality and morbidity, early recognition of SSTIs is challenging. Over half of patients are misdiagnosed at the early stages of disease, and the symptomatic triad of pain, erythema and swelling can be mistaken for the more common cellulitis or abscess.^11^ Delayed surgical decision, and unavailability of surgeons and facilities (such as operating theatre space) can further defer treatment.^12^^,^^13^ Interhospital transfer to a facility that can provide surgical debridement is also associated with increased mortality due to SSTIs.^14^ Essentially, delays in any aspect of SSTI management increases mortality but are unavoidable if the infection is recognized too late.

The aim of this study is to evaluate current approaches to managing SSTIs in the UK against “Surviving Sepsis Campaign” (SSC) and Sepsis 6 guidelines, and carry out a national retrospective analysis of SSTI cases including both adult and pediatric patients. Data from this study will be used to inform future research on SSTI treatment.

### Aims

Primary aim:

• To audit current practice for SSTIs against SSC and Sepsis 6 guidelines for adults and children.

Secondary aim:

• To provide a standardized approach to reporting SSTIs for future audits.
• To establish epidemiology of SSTIs in the UK.
• To identify key areas for future research.

## Materials/patients and methods

A national, multi-centered audit of NHS hospitals in England, Wales, Scotland and Northern Ireland will be carried out to achieve the above aims. Data collection will be retrospective to understand how documentation for both SSTIs and sepsis varies between sites, and can inform the methodology for later prospective studies. This study will follow a trainee collaborative model. Trainees in plastic surgery, adult intensive care and pediatric intensive care will be recruited from the three respective trainee collaborative networks: Reconstructive Surgery Trials Network (RSTN), Trainee Research in Intensive Care (TRIC) and Paediatric Intensive Care Audit Network (PICANet). Trainees from other surgical specialties are also welcome. In order to participate, each site ideally will require at least one surgical and one ICU collaborator to form a “collaborator unit”. From each site, trainees or consultants willing to act as the local lead will be asked to contact the study team to express interest and register their site. The study will be piloted at several sites *a priori* to ensure that accurate and reliable data will be efficiently collected.

Local leads at each site would need to register the audit locally and gain local Caldicott approval. They will receive guidance on local audit registration and approval, as well as on the data collection process. The study will begin in May 2025.

### Inclusion/Exclusion criteria

All patients who were treated for a severe necrotizing soft tissue infection (including necrotizing fasciitis [ICD-10 M72.6], necrotizing myositis and Fournier’s gangrene [ICD-10 N49.3]) and admitted to ICU (previous 12 months for adult patients, previous 5 years for pediatric patients) are eligible for the study.

Any patients treated solely for cellulitis (non-necrotizing) will be excluded. Patients who were not admitted to ICU will be excluded.

### Data collection

Routinely collected anonymized data will be uploaded onto REDCap, hosted at the Kennedy Institute of Rheumatology, University of Oxford. REDCap is a secure online platform created to store data for research. Each patient is assigned a REDCap ID, and no patient identifiable data will be held centrally.

Collaborators will be asked to keep an excel spreadsheet linking patient ID to their REDCap number to allow data validation checks later on. This spreadsheet should be stored on the local hospital’s secure server according to local IT policies.

Collaborators will be asked for each patient they upload onto REDCap to collect data on patient demographics, clinical presentation, investigations, and management in surgery and intensive care. A full list of required data fields are available in the supplementary material (Supplementary material 1 and 2).

### Data validation

After the data collection period, approximately 5 % of data at each site will be chosen for data validation. The local lead at each site will be asked to validate the selected proportion of the data at their site.

### Data analysis

As this is an exploratory study, no power calculation will be done for sample size. All data collected on REDCap will be exported to a statistical software platform. All captured data will undergo descriptive analysis. Dichotomous, categorical and short ordinal data will be summarized by counts and percentages. Long ordinal and continuous data will be summarized by an appropriate mean with 95 % confidence interval. Anonymized data will be made publicly available once the study is completed and published.

### Authorship

Collaborator units who submitted all cases over the time period, as confirmed by the consultant lead (either adult, pediatric or both) will be eligible for Pubmed citable collaborator status. They will receive a certificate that demonstrates their contribution to the study, and be Pubmed cited for subsequent publications related to this study. Collaborator units who contributed to data collection but did not reach the threshold will be named under acknowledgments and receive a certificate of participation. Sites will be required to submit a minimum number of cases to be eligible.

### Timeline

The study is currently recruiting collaborators. Data collection has begun in June 2025. We anticipate completion of both recruitment and data collection by 30th November 2025. Estimated completion of data analysis is 31st January 2026.

## Discussion

The SENSEI study will audit adherence to SSC and Sepsis 6 guidelines for SSTI patients. The audit findings will inform clinicians on current practices for sepsis management, identifying key areas for improving clinical practice and for future research. Improved awareness of SSTIs may allow clinicians to recognize these infections early. However, awareness may be limited due to their rare incidence, reflected in the lack of epidemiological data.

The World Society of Emergency Surgery (WSES) recently updated their guidelines on skin and soft tissue infections, which incorporates recent adult SSC guidelines.^15^ On the other hand, international guidelines for pediatric patients have not been established as pediatric cases are even rarer than adults, reported to be 0.293 cases per 100,000 population compared to 0.4–0.53 in adults.^16^

While there is a general consensus regarding treatment principles for these infections, there is lack of high-quality evidence^17^ - the rarity of SSTIs means that the mainstay of current research relies on small single-centered studies. A Cochrane review identified three randomized controlled trials (RCTSs) assessed effects of medical treatment for SSTIs, of which all three has high risk of attrition bias, and one was not blinded.^17^ The rare incidence of SSTIs means single-centered studies struggle to gather a large enough population size to generate meaningful results which may be resolved by multi-centered collaboration.^18^ Existing studies also vary greatly in how they measure patient outcomes.^19^ There has been no significant progression in our understanding of SSTIs in recent years. A multi-centered national study can address some of these issues by increasing the sample size and improving external validity. Designing a standardized form for reporting SSTIs allows for comparison between sites and facilitates a prospective cohort study in a national setting.

Since sepsis is present during these infections, current practices in sepsis management can be studied alongside SSTI management. In England and Wales, it was reported that sepsis was the underlying cause of 3770 deaths and a contributing factor for 25,452 deaths.^20^ Worldwide, sepsis is a leading cause of death with 11 million sepsis-related deaths, representing nearly 20 % of all global deaths.^21^ To reduce sepsis mortality and morbidity, the global initiative SSC published guidelines for sepsis management for adult and pediatric populations.^22^^,^^23^ These guidelines may address variations in sepsis management between hospitals.^24^, ^25^, ^26^ Furthermore, a national UK audit on Sepsis 6 care bundle showed that on average most of Sepsis 6 was not achieved within the first hour for the majority of septic patients.^27^ Evaluating management of sepsis in addition to SSTIs would provide an update on clinical practice and compliance with both SSC and Sepsis 6.

SSTIs also have substantial associated healthcare costs which highlight the importance of further study in this area. An Australian study in 2005 found a mean inpatient cost of managing necrotizing fasciitis of 64,517 AUD (£27,050 in the year of publication), not including future outpatient costs such as physical therapy or prosthetics and potential scar or revision surgery.^28^ A significant proportion of this total cost is due to the necessity for patients with SSTI to be managed in a critical care environment due to the presence of sepsis. Although there are no published data regarding predicted costs of ICU management for SSTIs specifically, baseline ICU costs in the UK have been estimated at around £1600,^29^ and median duration of ICU stay for necrotizing 8 days for this type of infection.^30^

To our knowledge, the SENSEI study will be the first UK multi-centered audit for SSTIs for both adult and pediatric populations. Multi-centered studies for SSTI patients exist in other countries but findings may not be transferable to the UK population.^31^, ^32^, ^33^ This study will establish a network of clinicians who will support and recruit patients for future high quality research in SSTIs, as well as providing a surveillance system for monitoring these infections.

## Strengths and limitations

### Strengths

• First UK multi-centre study of SSTIs, allowing for larger sample size and greater external validity than previous single-centre studies, which are limited by the relatively rarity of SSTIs.
• Evaluation of concordance with Sepsis 6/Surviving Sepsis Campaign recommendations in cases of SSTI.
• Forms an important baseline in SSTI epidemiology that can be used to standardize future data collection.
• Results can be utilized to inform future prospective research

## Limitations

• Standard limitations of retrospective observational data analysis including selection and misclassification bias, and the presence of missing data.

## Ethics and dissemination

### Ethical approval and informed consent

As this is an audit of routinely collected data and does not influence patient management, formal documentation of consent and HRA/NHS REC approval are not required for this study. (Supplementary material 3)

### Dissemination

Findings of the study will be disseminated via future conference presentations and a peer-reviewed open access paper. This study will inform future research pertaining to SSTI management.

### Patient and public involvement

Neither patients nor the public were involved in the design, however this audit forms the basis for further potential work based on key areas identified to improve outcomes in severe necrotizing soft tissue infection, which could involve both patients and the public.

## Declaration of competing interest

None.

## Appendix Supplementary materials

Supplementary material 1. REDCap data collection form for adult patients. Image, application 1

Supplementary material 2. REDCap data collection form for pediatric patients. Image, application 2

Supplementary material 3. Confirmation that this study does not require a review by the NHS Research Ethics Committee (REC). Image, application 3
