# Supplementary material for: Severe necrotizing soft tissue infections (SENSEI) study: Protocol for a multi-centered audit
Source: JPRAS Open. 2025 Nov 15;48:331–6. doi: 10.1016/j.jpra.2025.11.016 (PMC12769394; doi:10.1016/j.jpra.2025.11.016)
Supplement: Supplementary file 1 — Supplementary material 1. REDCap data collection form for adult patients. [file mmc1.pdf]

SENSEI Adults

Record ID ______________________

Patient Demographics

Number nights admitted in secondary care, including nights in ICU (please input number): ______________________

Number of nights admitted in ICU (please input number): ______________________

If septic, was patient admitted to ITU within 6 hours? (since arrival at ED or since deterioration at the ward)
- ◯ Yes
- ◯ No
- ◯ Not applicable - was not septic

Age of patient (years): ______________________

Sex at birth:
- ◯ Male
- ◯ Female

Patient Co-morbidities

Co-morbidities of patient (select one or more):

Risk factors that increase MRSA/MDRO risk

- Prior history of MRSA/MDRO or colonisation
- Recent IV antibiotics
- Recent broad spectrum antibiotic use (within last 90 days)
- History of recurrent skin infections or chronic wounds
- Presence of invasive devices
- Haemodialysis
- Recent hospital admission (especially if abroad in last 90 days).

- ☐ Respiratory
- ☐ Cardiac
- ☐ Renal
- ☐ Neurological
- ☐ Learning/Intellectual disability
- ☐ Gastroenterological
- ☐ Liver disease
- ☐ Known immune co-morbidity
- ☐ Haematological
- ☐ Diabetes mellitus type 1
- ☐ Diabetes mellitus type 2
- ☐ Other endocrinological (besides diabetes mellitus)
- ☐ BMI >30
- ☐ Dermatological
- ☐ Vascular
- ☐ Intravenous drug use
- ☐ High risk of MRSA (methicillin-resistant staphyloccocus aureus)
- ☐ High risk of MDRO (multi-drug resistant organism)

Does patient have any of the following? Steroids, chemotherapy or known immunodeficiency?
- ◯ Yes
- ◯ No

Was the patient on a SGLT-2 inhibitor? (e.g. dapagliflozin)
- ◯ Yes
- ◯ No

Patient Presentation

How was infection acquired?

- ◯ Community-acquired
- ◯ Hospital-acquired (not postoperative)
- ◯ Hospital-acquired (postoperative)

If community-acquired, what was length of fever prior to diagnosis (enter hours):

____

Patient had prior history of (select one or more):

- ☐ Sore throat
- ☐ Strep throat
- ☐ Coryzal symptoms

Number of separate presentations to medical teams prior to diagnosis:

Examples of presentation to medical teams include GP, paramedics, out of hour units, emergency department.

____

If infection was hospital acquired, how many times was the initial presentation raised to the medical team?

____

Site of severe soft tissue infection:

- ☐ Lower limb
- ☐ Perineal / Genital
- ☐ Abdomen / Thorax
- ☐ Upper limb
- ☐ Head and neck

Other presenting features (select one or more):

- ☐ Severe out-of-proportion pain beyond affected areas
- ☐ Highest temperature within 4 hours of presentation
- ☐ GI symptoms (vomiting or diarrhoea)
- ☐ Severe muscle pain
- ☐ Oral mucositis
- ☐ Conjunctivitis
- ☐ Rash or other skin changes

Define skin changes present:

- ☐ Erythema
- ☐ Fixed staining
- ☐ Purpura
- ☐ Blistering
- ☐ Pallor
- ☐ Oedema
- ☐ Crepitus

SOFA score - Respiratory Parameters

Intubated and ventilated during ICU admission?

- ◯ Yes
- ◯ No

PaO2 on diagnosis:

____

FiO2 on diagnosis:

____

PaO2 at its worst:

____

FiO2 at worst PaO2: ____________________

SpO2 on diagnosis: ____________________

SpO2 at its worst: ____________________

FiO2 at worst SpO2: ____________________

SOFA Score - Cardiovascular Parameters

Highest dopamine infusion rate (microgram/kg/min) in first hour of admission:
(Enter '0' if not used) ____________________

Highest adrenaline infusion rate (microgram/kg/min) in first hour of admission:
(Enter '0' if not used) ____________________

Highest noradrenaline infusion rate (microgram/kg/min) in first hour of admission:
(Enter '0' if not used) ____________________

Other vasoactive drug used in first hour of admission:
- ◯ Yes
- ◯ No

Other vasoactive drug used in ICU admission: ____________________

Worst lactate in first hour of presentation: ____________________

Worst lactate during ICU stay: ____________________

Worst MAP (mean arterial pressure) in first hour of presentation: ____________________

(If cardiac arrest occurred, please enter '0')

Worst MAP (mean arterial pressure) during ICU stay: ____________________

(if cardiac arrest occurred, please enter '0')

Was initial target of 65 mmHG MAP achieved with vasopressors?
- ◯ Yes
- ◯ No

If ongoing requirement for vasopressor therapy, were IV corticosteroids used?
- ◯ Yes
- ◯ No

SOFA score - Haematology Parameters

Platelet levels at presentation (x10^9/ml) ______________________

Worst platelet levels during ICU stay (x10^9/ml) ______________________

Was a platelet tranfusion(s) given during ICU stay?
- ◯ Yes
- ◯ No

What was Prothrombin Time (PT) measured at presentation? (Leave blank if not measured) ______________________

Worst PT during ICU stay? (Leave blank if not measured) ______________________

What was INR measured at presentation? (Leave blank if not measured) ______________________

Worst INR during ICU stay? (Leave blank if not measured) ______________________

What was D-dimer measured at presentation? (Leave blank if not measured) ______________________

Worst D-dimer during ICU stay? (Leave blank if not measured) ______________________

What was fibrinogen at presentation? (Leave blank if not measured) ______________________

Worst fibrinogen during ICU stay? (Leave blank if not measured) ______________________

Was FFP (fresh frozen plasma) or cryoprecipitate given during ICU stay?
- ☐ FFP
- ☐ Cryoprecipitate
- ☐ Neither

SOFA Score - Neurological Assessment

What was GCS (glasgow coma scale) score on presentation? ______________________

What were pupil reactions on presentation?
- ◯ Bilaterally normal
- ◯ Fixed unilaterally
- ◯ Fixed bilaterally
- ◯ Pupils responding to light but asymmetric

NICE sepsis guidelines

On presentation, did patient have any of the following:	☐ Recent trauma/surgery/invasive procedure (in past 6 weeks)<br>☐ Indwelling lines/broken skin
Which screening tool was used for sepsis?	◯ qSOFA<br>◯ SIRS<br>◯ NEWS2<br>◯ MEWS<br>◯ T&T<br>◯ Other<br>◯ None
If other scoring system for sepsis was used, please state name of scoring system:	__________________________________
What was the score for [score_status] screening tool?	__________________________________
Were the following sepsis red flags present on presentation?	☐ New altered mental state<br>☐ Respiratory rate 25 or more<br>☐ New oxygen requirement (FiO2 40% or more) to maintain SpO2 >92% (or >88% for COPD)<br>☐ Systolic blood pressure 90mmHg or less, or 40mmhg less than normal.<br>☐ Heart rate >130<br>☐ No urine output in previous 18 hours<br>☐ If catheterised - passed less than 0.5 ml/kg/hr<br>☐ Non-blanching rash/mottled/ashen/cyanosis
Were any of the following present on presentation?	☐ Abnormal cap refill<br>☐ Temp >38 or < 36<br>☐ None of above

Timing of sepsis management

When Fournier's Gangrene was suspected, were SLGT-2 inhibitors stopped?	◯ Yes<br>◯ No
State time of presentation:	__________________________________
When did triage take place? (State hours since time of presentation)	__________________________________
Grade of clinician who recognised severe soft tissue infection:	◯ Paramedic<br>◯ Nurse<br>◯ F1<br>◯ SHO<br>◯ Registrar<br>◯ Consultant
When did senior doctor review take place? (State hours since time of presentation)	__________________________________

Initial antibiotics given:	☐ Amoxicillin<br>☐ Co-Amoxiclav<br>☐ Clarithromycin<br>☐ Flucloxacillin<br>☐ Gentamicin<br>☐ Metronidazole<br>☐ Piperacillin/Tazobactam ("Piptaz", "Tazocin")<br>☐ Clindamycin<br>☐ Linezolid<br>☐ Other
If other antibiotic, please specify:	__________________________________
Timing of antibiotic administration (start of infusion):	◯ Within 1 hour (of recognition of sepsis)<br>◯ Within 3 hours (of recognition of sepsis)<br>◯ More than 3 hours (of recognition of sepsis)
IV fluids given:	◯ Balanced crystalloid (e.g. plasmalyte, Hartmann's, Ringer's lactate)<br>◯ 0.9% saline<br>◯ Starch<br>◯ Albumin<br>◯ Other
Timing of first fluid bolus, from presentation (in minutes):	__________________________________
Total bolus fluids (excluding blood products) given in first 24 hours (in litres):	__________________________________
Were any of the following used to guide resuscitation?	☐ Dynamic measures (e.g. passive leg raising & cardiac output, fluid challenges against stroke volume/systolic pressure/pulse pressure, increase in SV in response to change in intrathoracic pressure)<br>☐ Lactate<br>☐ Cap refill
Timing of first vasoactive medication, since presentation (in minutes):	__________________________________
Did patient have a urinary catheter within the first hour of sepsis recognition?	◯ Yes<br>◯ No

What were the following blood results on presentation?:

Haemoglobin (g/dl):	__________________________________
White blood cell count (x10^9 cells/ml):	__________________________________
Neutrophils (x10^9 cells/ml):	__________________________________
Lymphocytes (x10 cells/ml):	__________________________________

Sodium (mmol/l): __________________________________

Potassium (mmol/l): __________________________________

Urea (mmol/l): __________________________________

Creatinine (micromol/l): __________________________________

CRP (mg/l): __________________________________

Alanine transferase (IU/l): __________________________________

Bilirubin (mg/dl): __________________________________

Creatine kinase (mmol/l): __________________________________

Glucose (mmol/l): __________________________________

Procalcitonin: __________________________________

What was the worst result during patient's ICU stay for the following bloods?:

Haemoglobin (g/dl): __________________________________

White blood cell count (x10^9 cells/ml): __________________________________

Neutrophils (x10^9 cells/ml): __________________________________

Lymphocytes (x10 cells/ml): __________________________________

Sodium (mmol/l): __________________________________

Potassium (mmol/l): __________________________________

Urea (mmol/l): __________________________________

Creatinine (micromol/l): __________________________________

CRP (mg/l): ______________________

Alanine transferase (IU/l): ______________________

Bilirubin (mg/dl): ______________________

Creatine kinase (mmol/l) - if unknown, please leave blank: ______________________

Glucose (mmol/l): ______________________

Procalcitonin: ______________________

Blood cultures

Were blood cultures taken within the first hour upon sepsis recognition?
◯ Yes
◯ No

Were blood cultures taken before antibiotic administration?
◯ Yes
◯ No

What were the blood culture results? (state negative if no organisms found) ______________________

Other ICU management

Did patient receive VTE prophylaxis?
◯ Yes
◯ No

Did patient have any of the complications of inotropic support?:
☐ Ischaemia / Necrosis
☐ Arrhythmia
☐ Stroke
☐ No complications

Was renal filtration required?
◯ Yes
◯ No

Was IV Immunoglobulin given?
◯ Yes
◯ No

Surgical Management

Do local pathways exist for managing severe soft tissue infections?	◯ Yes<br>◯ No
Was finger sweep test done?	◯ Yes<br>◯ No
Was any imaging used for diagnosis?:	☐ XR<br>☐ MRI<br>☐ CT<br>☐ None
Did imaging delay management in any way?	◯ Yes<br>◯ No
When did decision to do surgery take place? (State hours since time of presentation)	______________________
Was patient transferred to another hospital prior to surgical debridement?	◯ Yes<br>◯ No

Initial debridement

Did debridement occur within 12 hours of presentation?	◯ Yes<br>◯ No
Did debridement occur within 6 hours of presentation?	◯ Yes<br>◯ No
If not done within 6hrs/12hrs, select reason(s) for delay:	☐ Delayed diagnosis<br>☐ Misdiagnosis<br>☐ Theatre capacity<br>☐ Unavailability of surgeon<br>☐ Other
How many hours after presentation did debridement take place?	______________________
Did infection / necrosis involve muscle?	◯ Yes<br>◯ No
Which specialty performed/led the initial debridement?	◯ General surgey<br>◯ Orthopaedics<br>◯ Plastic surgery<br>◯ Other

2nd relook surgery

Did second relook surgery occur within 24 hours after initial debridement?	◯ Yes<br>◯ No
If no, give a reason:	______________________________

Other operative variables

Was amputation required? (either in initial operation or subsequent procedures).	◯ Yes<br>◯ No
If amputation occurred, state the limb and level amputated:	______________________________
Were samples taken during surgical debridement?	◯ Yes<br>◯ No
If yes, what were the microbiology results?	☐ Polymicrobial (i.e. Type 1)<br>☐ Streptococcus spp or Staph Aureus (Type 2)<br>☐ Vibrio spp or Aeromonas species (Type 3)<br>☐ Fungal infection e.g. Candida spp, Zygomycetes (Type 4)<br>☐ Other<br>☐ Negative
If other, please specify micro results	______________________________
Did histology show evidence of acute necrotising fasciitis/myositis?	◯ Yes<br>◯ No<br>◯ No histology results
Was negative pressure wound therapy used post-debridement?	◯ Yes<br>◯ No
State total number of debridements that occurred:	______________________________
After debridement, how was the wound reconstructed?	☐ No reconstruction<br>☐ Direct closure<br>☐ Skin graft<br>☐ Dermal substitute with skin graft<br>☐ Local flap<br>☐ Free flap<br>☐ Other
Did patient have recurring wound issues after acute phase of infection?	◯ Yes<br>◯ No
Did patient survive beyond 3 months of initial presentation?	◯ Yes<br>◯ No
