# Supplementary material for: Severe necrotizing soft tissue infections (SENSEI) study: Protocol for a multi-centered audit
Source: JPRAS Open. 2025 Nov 15;48:331–6. doi: 10.1016/j.jpra.2025.11.016 (PMC12769394; doi:10.1016/j.jpra.2025.11.016)
Supplement: Supplementary file 2 — Supplementary material 2. REDCap data collection form for pediatric patients. [file mmc2.pdf]

SENSEI Paeds

Record ID ____________________

Record ID ____________________

Patient Demographics

Number nights admitted in secondary care, including nights in ICU (please input number): ____________________

Number of nights admitted in ICU (please input number): ____________________

Required transfer to a centre with PICU?
- ◯ Yes
- ◯ No

If septic, was patient admitted to ITU within 6 hours? (since arrival at ED or since deterioration at the ward)
- ◯ Yes
- ◯ No
- ◯ Not applicable - was not septic

Age of patient (years): ____________________

Sex at birth:
- ◯ Male
- ◯ Female

Patient Co-morbidities

Co-morbidities of patient (select one or more):

Risk factors that increase MRSA/MDRO risk

- Prior history of MRSA/MDRO or colonisation
- Recent IV antibiotics
- Recent broad spectrum antibiotic use (within last 90 days)
- History of recurrent skin infections or chronic wounds
- Presence of invasive devices
- Haemodialysis
- Recent hospital admission (especially if abroad in last 90 days).

- ☐ Prematurity
- ☐ Respiratory
- ☐ Cardiac
- ☐ Renal
- ☐ Neurological
- ☐ Learning/Intellectual disability
- ☐ Gastroenterological
- ☐ Liver disease
- ☐ Known immune co-morbidity
- ☐ Haematological
- ☐ Diabetes mellitus type 1
- ☐ Diabetes mellitus type 2
- ☐ Other endocrinological (besides diabetes mellitus)
- ☐ BMI >30
- ☐ Dermatological
- ☐ Vascular
- ☐ Intravenous drug use
- ☐ High risk of MRSA (methicillin-resistant staphyloccocus aureus)
- ☐ High risk of MDRO (multi-drug resistant organism)

Does patient have any of the following? Steroids, chemotherapy or known immunodeficiency?
- ◯ Yes
- ◯ No

Was the patient on a SGLT-2 inhibitor? (e.g. dapagliflozin)
- ◯ Yes
- ◯ No

Patient Presentation

How was infection acquired?
- ◯ Community-acquired
- ◯ Hospital-acquired (not postoperative)
- ◯ Hospital-acquired (postoperative)

If community-acquired, what was length of fever prior to diagnosis (enter hours): ______________________

Patient had prior history of (select one or more):
- ☐ Sore throat
- ☐ Strep throat
- ☐ Coryzal symptoms

Number of separate presentations to medical teams prior to diagnosis: ______________________

Examples of presentation to medical teams include GP, paramedics, out of hour units, emergency department.

If infection was hospital acquired, how many times was the initial presentation raised to the medical team? ______________________

Site of severe soft tissue infection:
- ☐ Lower limb
- ☐ Perineal / Genital
- ☐ Abdomen / Thorax
- ☐ Upper limb
- ☐ Head and neck

Other presenting features (select one or more):
- ☐ Severe out-of-proportion pain beyond affected areas
- ☐ Highest temperature within 4 hours of presentation
- ☐ GI symptoms (vomiting or diarrhoea)
- ☐ Severe muscle pain
- ☐ Oral mucositis
- ☐ Conjunctivitis
- ☐ Rash or other skin changes

Define skin changes present:
- ☐ Erythema
- ☐ Fixed staining
- ☐ Purpura
- ☐ Blistering
- ☐ Pallor
- ☐ Oedema
- ☐ Crepitus

Phoenix Sepsis/pSOFA Score - Respiratory Parameters

Intubated and ventilated during ICU admission?
- ◯ Yes
- ◯ No

PaO2 on diagnosis: ______________________

FiO2 on diagnosis: ______________________

PaO2 at its worst: ______________________

FiO2 at worst PaO2: ______________________

SpO2 on diagnosis: ______________________

SpO2 at its worst: ______________________

FiO2 at worst SpO2: ______________________

Phoenix Sepsis/pSOFA Score - Cardiovascular Parameters

Number of vasoactive medications in first hour of presentation (enter '0' if not used) ______________________

Maximum number of vasoactive medications during PICU stay (enter '0' if not used) ______________________

Worst lactate in first hour of presentation: ______________________

Worst lactate during PICU stay: ______________________

Worst MAP (mean arterial pressure) in first hour of presentation:

(If cardiac arrest occurred, please enter '0') ______________________

Worst MAP (mean arterial pressure) during PICU stay: (If cardiac arrest occurred, please enter '0') ______________________

Was initial target MAP achieved with vasopressors?
- ◯ Yes
- ◯ No

If ongoing requirement for vasopressor therapy, were IV corticosteroids used?
- ◯ Yes
- ◯ No

Phoenix Sepsis/pSOFA Score - Haematology Parameters

Platelet levels at presentation (x10^9/ml) ______________________

Worst platelet levels during ICU stay (x10^9/ml) ______________________

Was a platelet tranfusion(s) given during ICU stay?
- ◯ Yes
- ◯ No

What was Prothrombin Time (PT) measured at presentation? (Leave blank if not measured) ______________________

Worst PT during ICU stay? (Leave blank if not measured) ______________________

What was INR measured at presentation? (Leave blank if not measured) ______________________

Worst INR during ICU stay? (Leave blank if not measured) ______________________

What was D-dimer measured at presentation? (Leave blank if not measured) ______________________

Worst D-dimer during ICU stay? (Leave blank if not measured) ______________________

What was fibrinogen at presentation? (Leave blank if not measured) ______________________

Worst fibrinogen during ICU stay? (Leave blank if not measured) ______________________

Was FFP (fresh frozen plasma) or cryoprecipitate given during ICU stay?
- ☐ FFP
- ☐ Cryoprecipitate
- ☐ Neither

Phoenix Sepsis/pSOFA Score - Neurological Assessment

What was GCS (glasgow coma scale) score on presentation? ______________________

What were pupil reactions on presentation?
- ◯ Bilaterally normal
- ◯ Fixed unilaterally
- ◯ Fixed bilaterally
- ◯ Pupils responding to light but asymmetric

NICE sepsis guidelines

On presentation, did patient have any of the following:
- ☐ Recent trauma/surgery/invasive procedure (in past 6 weeks)
- ☐ Indwelling lines/broken skin

Which paediatric creening tool was used for sepsis?
- ◯ Phoenix
- ◯ SIRS
- ◯ NEWS2
- ◯ PEWS
- ◯ Other
- ◯ None

If other scoring system for sepsis was used, please state name of scoring system: ______________________

What was the score for [score_status] screening tool? ______________________

Were the following sepsis red flags present on presentation?	☐ Doesn't wake when roused<br>☐ New altered mental state<br>☐ Respiratory rate 25 or more<br>☐ SpO2 < 90% on air or increased O2 requirement<br>☐ Heart rate >130<br>☐ Weak, high pitched, or continuous cry<br>☐ Non-blanching rash/mottled/ashen/cyanosis<br>☐ Looks very unwell to a professional<br>☐ Temperature above 38 or below 36 degrees centigrade

Timing of sepsis management

When Fournier's Gangrene was suspected, were SLGT-2 inhibitors stopped?	◯ Yes<br>◯ No
State time of presentation:	__________________________________
When did triage take place? (State hours and minutes since time of presentation)	__________________________________
Grade of clinician who recognised severe soft tissue infection:	◯ Paramedic<br>◯ Nurse<br>◯ F1<br>◯ SHO<br>◯ Registrar<br>◯ Consultant
When did senior doctor review take place? (State hours and minutes since time of presentation)	__________________________________
Timing of first IV access	◯ Within 1 hour (of recognition of sepsis)<br>◯ Within 3 hours (of recognition of sepsis)<br>◯ More than 3 hours (of recognition of sepsis)
Initial antibiotics given:	☐ Amoxicillin<br>☐ Co-Amoxiclav<br>☐ Clarithromycin<br>☐ Flucloxacillin<br>☐ Gentamicin<br>☐ Metronidazole<br>☐ Piperacillin/Tazobactam ("Piptaz", "Tazocin")<br>☐ Clindamycin<br>☐ Linezolid<br>☐ Other
If other antibiotic, please specify:	__________________________________
Timing of antibiotic administration (start of infusion):	◯ Within 1 hour (of recognition of sepsis)<br>◯ Within 3 hours (of recognition of sepsis)<br>◯ More than 3 hours (of recognition of sepsis)

IV fluids given:

- ◯ Balanced crystalloid (e.g. plasmalyte, Hartmann's, Ringer's lactate)
- ◯ 0.9% saline
- ◯ Starch
- ◯ Albumin
- ◯ Other

Timing of first fluid bolus, from presentation (in minutes): ______________________

Total bolus fluids (excluding blood products) given in first 24 hours (in ml/kg): ______________________ (ml/kg)

Were any of the following used to guide resuscitation?

- ☐ Dynamic measures (e.g. passive leg raising & cardiac output, fluid challenges against stroke volume/systolic pressure/pulse pressure, increase in SV in response to change in intrathoracic pressure)
- ☐ Lactate
- ☐ Cap refill

Timing of first vasoactive medication, since presentation (in minutes): ______________________

What were the following blood results on presentation?:

Haemoglobin (g/dl): ______________________

White blood cell count (x10^9 cells/ml): ______________________

Neutrophils (x10^9 cells/ml): ______________________

Lymphocytes (x10 cells/ml): ______________________

Sodium (mmol/l): ______________________

Potassium (mmol/l): ______________________

Urea (mmol/l): ______________________

Creatinine (micromol/l): ______________________

CRP (mg/l): ______________________

Alanine transferase (IU/l): ______________________

Bilirubin (mg/dl): __________________________________

Creatine kinase (mmol/l): __________________________________

Glucose (mmol/l): __________________________________

Procalcitonin: __________________________________

What was the worst result during patient's ICU stay for the following bloods?:

Haemoglobin (g/dl): __________________________________

White blood cell count (x10^9 cells/ml): __________________________________

Neutrophils (x10^9 cells/ml): __________________________________

Lymphocytes (x10 cells/ml): __________________________________

Sodium (mmol/l): __________________________________

Potassium (mmol/l): __________________________________

Creatinine (micromol/l): __________________________________

Urea (mmol/l): __________________________________

CRP (mg/l): __________________________________

Alanine transferase (IU/l): __________________________________

Bilirubin (mg/dl): __________________________________

Creatine kinase (mmol/l) - if unknown, please leave blank: __________________________________

Glucose (mmol/l): __________________________________

Procalcitonin: __________________________________

Blood cultures

Were blood cultures taken within the first hour upon sepsis recognition?
◯ Yes
◯ No

Were blood cultures taken before antibiotic administration?
◯ Yes
◯ No

What were the blood culture results? (state negative if no organisms found)
____

Other ICU Management

Did patient have any of the complications of inotropic support?:
☐ Ischaemia / Necrosis
☐ Arrhythmia
☐ Stroke

Was renal filtration required?
◯ Yes
◯ No

Was IV immunoglobulin given?
◯ Yes
◯ No

Surgical Management

Do local pathways exist for managing severe soft tissue infections?
◯ Yes
◯ No

Was finger sweep test done?
◯ Yes
◯ No

Was any imaging used for diagnosis?:
☐ XR
☐ MRI
☐ CT
☐ None

Did imaging delay management in any way?
◯ Yes
◯ No

When did decision to do surgery take place? (State hours and minutes since time of presentation)
____

Was patient transferred to another hospital prior to surgical debridement?
◯ Yes
◯ No

Initial debridement

Did debridement occur within 12 hours of presentation?
◯ Yes
◯ No

Did debridement occur within 6 hours of presentation?
◯ Yes
◯ No

If not done within 6hrs/12hrs, select reason(s) for delay:	☐ Delayed diagnosis<br>☐ Misdiagnosis<br>☐ Theatre capacity<br>☐ Unavailability of surgeon<br>☐ Other
How many hours after presentation did debridement take place?	__________________________
Did infection / necrosis involve muscle?	◯ Yes<br>◯ No
Which specialty performed/led the initial debridement?	◯ General surgey<br>◯ Orthopaedics<br>◯ Plastic surgery<br>◯ Other

2nd relook surgery

Did second relook surgery occur within 24 hours after initial debridement?	◯ Yes<br>◯ No
If no, give a reason:	__________________________

Other operative variables

Was amputation required?	◯ Yes<br>◯ No
If amputation occurred, state the limb and level amputated:	__________________________
Were samples taken during surgical debridement?	◯ Yes<br>◯ No
If yes, what were the microbiology results?	☐ Skin flora<br>☐ Polymicrobial (i.e. Type 1)<br>☐ Streptococcus spp or Staph Aureus (Type 2)<br>☐ Vibrio spp or Aeromonas species (Type 3)<br>☐ Fungal infection e.g. Candida spp, Zygomycetes (Type 4)<br>☐ Negative
If other, please specify micro results	__________________________
Did histology show evidence of acute necrotising fasciitis/myositis?	◯ Yes<br>◯ No<br>◯ No histology results
Was negative pressure wound therapy used post-debridement?	◯ Yes<br>◯ No
State total number of debridements that occurred:	__________________________

After debridement, how was the wound reconstructed?	☐ No reconstruction<br>☐ Direct closure<br>☐ Skin graft<br>☐ Dermal substitute with skin graft<br>☐ Local flap<br>☐ Free flap<br>☐ Other
Did patient have recurring wound issues after acute phase of infection?	◯ Yes<br>◯ No
Did patient survive beyond 3 months of initial presentation?	◯ Yes<br>◯ No
