# Supplementary material for: Severe necrotizing soft tissue infections (SENSEI) study: Protocol for a multi-centered audit
Source: JPRAS Open. 2025 Nov 15;48:331–6. doi: 10.1016/j.jpra.2025.11.016 (PMC12769394; doi:10.1016/j.jpra.2025.11.016)
Supplement: Supplementary file 3 — Supplementary material 3. Confirmation that this study does not require a review by the NHS Research Ethics Committee (REC). [file mmc3.pdf]

Medical Research Council

NHS Health Research Authority

Do I need NHS REC review?

To print your result with title and IRAS Project ID please enter your details below:

Title of your research:

Severe Necrotising Soft Tissue Infections (SENSEI) Study: protocol for a multi-centred audit

IRAS Project ID (if available):

You have answered **'No'** to the question "Is your study research" which indicates that **you do not need NHS REC review**.

This tool only considers whether NHS REC review is required, it does not consider whether other approvals are needed. You should check whether other approvals are required for your study.

Note: **Post Market Surveillance** is NOT usually considered research. However, there are some circumstances where NHS REC review may be required. Please follow the link below to start again and select YES at the first question to determine if your post market surveillance requires NHS REC review.

To understand how research is defined, please visit the **Is my study research?** decision tool.

Follow this link to start again.

Print This Page

NOTE: If using Internet Explorer please use browser print function.
